# Facile Fabrication of Hierarchical rGO/PANI@PtNi Nanocomposite via Microwave-Assisted Treatment for Non-Enzymatic Detection of Hydrogen Peroxide

**DOI:** 10.3390/nano9081109

**Published:** 2019-08-02

**Authors:** Fa-Gui He, Jia-Yi Yin, Gaurav Sharma, Amit Kumar, Florian J. Stadler, Bing Du

**Affiliations:** 1 College of Materials Science and Engineering, Shenzhen Key Laboratory of Polymer Science and Technology, Guangdong Research Center for Interfacial Engineering of Functional Materials, Nanshan District Key Lab for Biopolymers and Safety Evaluation, Shenzhen University, Shenzhen 518055, China; 2 Department of Optoelectronic Engineering, Shenzhen University, Shenzhen 518060, China

**Keywords:** polyaniline, platinum–nickel nanoparticles, reduced graphene oxide, nonenzymatic detection, hydrogen peroxide

## Abstract

A hierarchical composite based on the modified reduced graphene oxide with platinum-nickel decorated polyaniline nano-spheres (rGO/PANI@PtNi) was facilely prepared via microwave-assisted self-reduction for an application in nonenzymatic hydrogen peroxide (H_2_O_2_) detection. Compared to the pristine rGO, the composite exhibited a much tougher surface due to the stacking of conductive PANI nano-spheres on rGO sheets, leading to good dispersion of PtNi nanoparticles and a large active area. Furthermore, the multi-valance Ni^2+/3+^ in the PtNi particles effectively promoted the catalytic property of Pt sites and facilitated a superior electrochemical performance of PtNi alloy than that of neat Pt. Owing to the synergistic effect of the improved electrical conductivity and the promoted electrocatalytical property, the modified glassy carbon electrode (GCE) with rGO/PANI@PtNi nanocomposite displayed an outstanding electrochemical sensitivity towards H_2_O_2_ with a fast response time (<2 s), a wide linear range (0.1–126.4 mM), a low detection limit (0.5 µM), as well as a long-life stability for one week without obvious degradation. This novel strategy opens a novel and promising approach to design high performance sensors for H_2_O_2_ detection.

## 1. Introduction

Hydrogen peroxide (H_2_O_2_) is one of the common bleaching or cleaning agents and a powerful oxidant that is widely used in many industrial processes [1,2,3,4]. Unfortunately, H_2_O_2_ also has very negative effects on the cell proliferation by triggering several kinds of essential signaling proteins, which leads to potentially serious diseases in our bodies, such as cancer, infarction, and atherosclerosis [5]. This renders it crucially important to accurately detect the concentration level of H_2_O_2_ in both natural and artificial surroundings. Among all the established technologies, the electrochemical detection is widely regarded as an effective and economical method owing to its simple instrumentation, rapid response, as well as its high sensitivity and selectivity [5,6,7]. In general, the electrochemical sensors for H_2_O_2_ determination are classified into enzymatic and non-enzymatic sensors according to their active components. Compared to the enzyme-based sensors, the non-enzymatic counterparts are identified to possess several noticeable advantages, such as better reproducibility and stability as well as higher and broader responsibility [8,9,10]. In the recent decades, considerable effort and attention have been devoted to developing the non-enzymatic modified electrode sensor systems for H_2_O_2_ detection [11,12,13].

As one typical catalyst, platinum (Pt) presents excellent performance in many electrochemical reactions, including in the decomposition of H_2_O_2_ into aqua (H_2_O) and oxygen (O_2_) [14]. Particularly, Pt can maintain its high activity in a neutral environment, which is favorable for the requested conditions of H_2_O_2_ detection [15]. Amatore’s group microfabricated a platinum-black coated platinum (Pt/Pt-black) electrode that showed high sensitivity, allowing almost five decades of concentration with a detecting limitation down to 10 nM [16]. In many recent works, several alloys based on Pt have been verified to possess even better performance than pristine Pt with improved catalytical activity, anti-interference, and long-term duration originating from the specific interaction in the alloys [17,18,19,20]. However, such nano-catalysts with extremely high surface energy cannot directly modify the electrode alone but have to be used with an essential support material to obtain satisfactory dispersibility and well-controlled particle size for sensing application. Among many possibilities, graphene oxide (GO) has been widely accepted as one extraordinary candidate for immobilizing the precursor of the catalysts owing to its substantial lattice defects and chemical groups [21]. Furthermore, the subsequent reduced graphene oxide (rGO) is a typical good conductor for electrochemical sensors with several superiorities, e.g., large surface area, excellent electron transport, as well as wide potential window [17,22,23,24,25,26]. To date, a good performance of the modified electrode on the basis of graphene and Pt-based catalyst composites has been reported in a great deal of literature [17,22,27,28,29].

However, there is still significant room for improvement in, for example, the aggregation for both graphene sheets and catalytic particles. To overcome this issue, the surfactants were introduced onto the graphene surfaces to counter the agglomeration and the hydrophobic properties of both graphene and metal-nanoparticles, such as cetyl trimethyl ammonium bromide (CTAB), sodium dodecyl benzyl sulfate (SDBS), and sodium dodecyl sulfate (SDS) [17,30,31,32,33,34]. These surfactants can not only contribute to perfecting the dispersion of the nano-components but can also facilitate the size construability of the catalysts due to the amplified active area for anchoring the metallic precursor or particles. Nonetheless, it is worth noting that most of the surface active agents for the graphene-based sensors reported thus far are insulating or semi-conductive in nature. Very recently, a few works started to use conductive polymers as the surfactant, expecting to achieve the improvement for both the dispersibility and the electrochemical properties. Qian et al. [35] loaded flow-like Au nano-particles (diameter ≈ 80 nm) on polypyrrole/rGO (PPy/rGO) hybrid sheets via in-situ chemical oxidative polymerization. The modified glassy carbon electrode (GCE) based on this composite exhibited a good electrochemical performance for dopamine detection with remarkable sensitivity of 16.40 μA/μM, low limitation of detection (18.29 pM), and wide linear response range (0.1–5000 nM). Zheng et al. [23] employed PANI nano-fibers with a diameter of 100–200 nm as the support for copper particles (Cu) before depositing them on graphene sheets through sonication. In their case, the size of Cu nanoparticles was well controlled with a diameter of 2–4 nm in the presence of PANI fibers. Such size-controlled catalytic particles showed obvious contributions in the improved detecting behavior.

**Figure 1:**
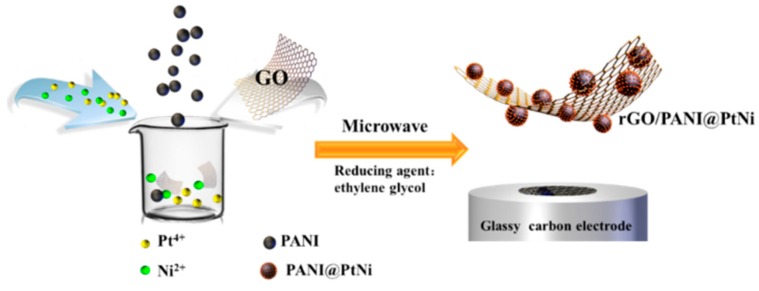
Scheme of fabricating the modified glassy carbon electrode (GCE) with platinum-nickel decorated polyaniline nano-spheres (rGO/PANI@PtNi) nanocomposite.

Herein, we fabricated a novel hierarchical nanocomposite combining stable aqueous polyaniline (PANI) nano-spheres and catalytical PtNi particles as well as rGO through a facile microwave treatment, which displayed a high sensitivity for quantitative H_2_O_2_ detection (Figure 1). PANI nano-spheres with an average diameter of 70 nm were employed for accelerating the electron transferring and also played as the surfactant in order to control the size of PtNi nanoparticles and improve the dispersibility of the PtNi particles. The catalytical nanoparticles were found to cover the PANI spheres without agglomeration. This unique morphology facilitated the interaction among all three active components and led to superior synergisms in the composite, allowing for improved electrochemical properties and superior detecting capability for H_2_O_2_.

## 2. Materials and Methods 

### 2.1. Materials and Apparatus

Graphene oxide (GO) was synthesized from pristine graphite (AlfaAesar, Tianjin, China) by following a modified Hummers method [36,37]. Polyvinyl alcohol (PVA, *M_w_ =* 27,000 g/mol, A.R., 99%), ethylene glycol (EG, A.R., 98%), ammonium persulphate (APS, A.R., 98.5%), and hydrogen peroxide (H_2_O_2_, A.R., 30 wt.% in water) were obtained from Macklin Reagent Company (Shanghai, China). Chloroplatinic acid hexahydrate (H_2_PtCl_6_·6H_2_O, 37.5 wt.% Pt) was obtained from Sigma-Aldrich (St. Louis, MO, USA). Potassium phosphate monobasic (KH_2_PO_4_, A.R., 99.5%), sodium phosphate dibasic dodecahydrate (Na_2_HPO_4_·12H_2_O, A.R., 99%), potassium chloride (KCl, A.R., 99.5%), and potassium ferricyanide (K_3_Fe(CN)_6_, A.R., 99.5%) were purchased from Aladdin Reagent Ltd. (Shanghai, China). N,N-dimethylformamide (DMF, A.R., 99%) and nickel acetate tetrahydrate (NiC_4_H_6_O_4_·4H_2_O, A.R., 99%) were obtained from Shanghai Chemical Factory (Shanghai, China). All chemicals were used as received without further purification. The water used throughout all experiments was ultrapure water from a Millipore system. Phosphate buffer saline (PBS) was prepared by mixing stock solutions of KH_2_PO_4_ and Na_2_HPO_4_. A fresh solution of H_2_O_2_ was prepared daily.

The samples were characterized by scanning electron microscopy (SEM, SU-70, Hitachi, Japan), transmission electron microscopy (TEM, JEM-1230, Nippon Tekno, Japan), high-resolution transmission electron microscopy (HRTEM, HT-7700, Hitachi, Japan), X-Ray diffraction (XRD, D8 Avance, Bruker, Karlsruhe, Germany), in-situ X-ray photoelectron spectroscopy (in-situ XPS, PHI 5000 VersaProbe II, ULVAC-*PHI*, Japan), Fourier transform infrared spectroscopy (FT-IR, Nicolet 6700, Thermofisher Scientific, Waltham, MA, USA), and Raman spectroscope (inVia Raman Microscope, Renishaw, UK). The conductivity of PANI powder was tested with the ST2722-SZ four probe resistivity measuring instrument (Suzhou Jingge Electronic Co., Ltd., Suzhou, China). All electrochemical experiments were carried out at room temperature using an IVIUM electrochemical workstation (CompactStat.e 10800, Ivium Technology, Eindhoven, Netherlands) with a conventional three-electrode system. Bare or modified GCE, Ag/AgCl electrode, and Pt wire were used as working electrode, reference electrode, and counter electrode, respectively. The interfacial charge transfer resistances for different modified surfaces were determined by electronic impedance spectroscopy (EIS) in the frequency range between 1 Hz and 1 MHz with a perturbation signal of 5 mV. For current-time experiments, 4 μL of Nafion (0.5%) was additionally cast on the surface of the rGO/PANI@PtNi modified GC electrode and dried before electrochemical experiments.

### 2.2. Synthesis of PANI Nano-Sphere

First, 2 mL aniline was added into 10 mL PVA solution (5 wt.% in distilled water) under magnetic stirring at room temperature for 0.5 h. Then, 2 mL of HCl solution (2 M) was added into the system, and the brown suspension subsequently appeared. After 0.5 h, 8 mL of APS solution (0.02 M) was added dropwise slowly, and then the system was under rigorous stirring for another 1 h at 5 °C. Afterwards, the suspension was kept at 4 °C for 24 h; the resulting substance was collected by centrifugation (5000 rpm, 10 min) and cleaned by repetitive washing with double-distilled water. Finally, the obtained PANI nanoparticles were purified by Soxhlet extraction and dried at 60 °C in vacuo.

### 2.3. Synthesis of rGO/PANI@PtNi Nanocomposite via Microwave Method

In a typical synthesis, 2 mL of GO-water suspension (2 mg/mL) was added into 2 mL EG. Then, sequentially, 1 mL of H_2_PtCl_6_ EG solution (77 mM), 1 mL of Ni(CHCOO)_2_ EG solution (228 mM), and 0.2 mL of PANI suspension (2 wt.% in deionized (DI) water) were added. After being sonicated for 2 min, the suspension was microwaved for 120 s (JOYN-H1C1 microwave oven, Shanghai Joyn Electronic Co., Ltd. China, power: 700 W). The product was isolated by centrifugation at 10,000 rpm for 15 min, followed by three washing/centrifugation cycles in water. The collected product was redispersed in 10 mL of ethanol by means of sonication, and colloidal suspension was obtained.

For better comparison, the individual components of this composite, namely neat rGO (the TEM image is shown in Supporting Information, Figure S1), platinum nanoparticles (the TEM image is shown in Figure S2), rGO/Pt (the TEM image is shown in Figure S3), rGO/PtNi (the TEM image is shown in Figure S4), and rGO/Pt/PANI (the SEM images are shown in Figure S5) were also prepared through almost the same process but without the addition of rGO, PANI, or without both PANI and metallic precursors.

### 2.4. Preparation of Modified GCE Electrode

The GCE (a disk with a diameter of 3 mm) was polished with 1, 0.5, and 0.3 mm alumina powder, respectively and then successively rinsed by sonication with ethanol and deionized water. The polished GCE was allowed to dry under a gentle nitrogen stream before use. Then, the modified GCE was fabricated by a simple casting method of dropping 5 μL of rGO/PANI@PtNi suspension (0.1 mg/mL, dispersed in ethanol) onto the cleaned GCE surface. The electrode was dried in the atmosphere before further characterization.

To be compared, rGO/GCE, Pt/GCE, rGO/Pt/GCE, rGO/PtNi/GCE, and rGO/ PANI@Pt/GCE electrodes were prepared through the same procedure.

## 3. Results

### 3.1. Characterization of PANI Nano-Spheres

**Figure 2:**
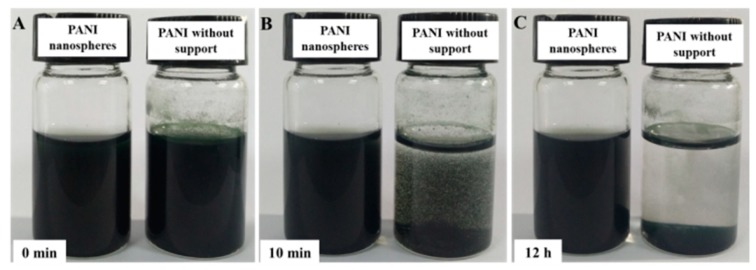
Photographs of aqueous PANI and pristine polyaniline dispersion (15 mL, 0.2 mg/mL): as ultra-sonicated (**A**); after leaving it undisturbed for 10 min (**B**); after leaving it undisturbed for 12 h (**C**).

It is known that PANI is an excellent conductive polymer and is widely used for electrochemical applications. However, the poor water dispersibility of PANI hampers its processability and performance. Introducing a water soluble polymer as a support during the polymerization is considered to be an effective solution to improve water dispersibility and mechanical properties of PANI [38,39]. In this work, we synthesized proton acid doped polyaniline hybrid in the presence of water soluble PVA as the support. As a reference, the pristine PANI was also prepared without PVA. As shown in Figure 2, PANI with PVA support maintained good dispersibility in water, even after standing for 12 h, while the pristine PANI started to aggregate just after 10 min and absolutely formed sediment after 12 h. Apparently, using PVA support can efficiently improve the dispersibility of PANI, which is expected to be beneficial to achieve good homogeneity for the ultimate composites.

The chemical structure of as-synthesized PANI was identified by FT-IR spectrum (Figure S6). The bands located at 3406 cm^−1^ and 1289 cm^−1^ corresponded to the stretching vibrations of N−H and C−N, respectively [40,41,42,43,44]. The appearance of these bands indicated the existence of the amide group from PANI [40,44,45]. Importantly, another strong band at 1122 cm^−1^ was associated with a high degree of electron delocalization and transfer in PANI, illustrating that the as-synthesized PANI was in the protonated emeraldine (EM) state [41,46,47,48,49]. This result was in accordance with the electrical conductivity of 4.9 s/cm for the received PANI powder determined by a standard four-probe technique.

### 3.2. Morphology and Structure of the Composites

**Figure 3:**
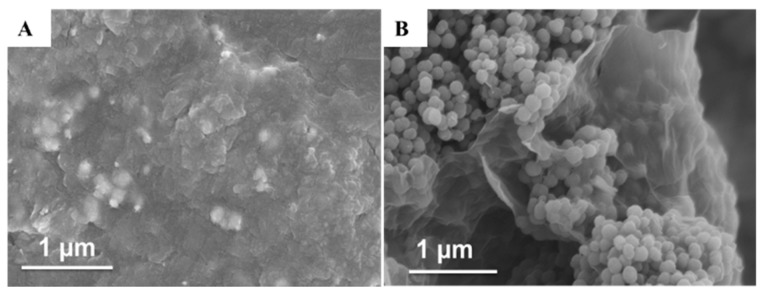
SEM images of rGO/PtNi (**A**) and rGO/PANI@PtNi (**B**).

Figure 3 gives the SEM images of rGO/PtNi and rGO/PANI@PtNi, respectively. rGO/PtNi exhibited as a flat-sheet, where the surface was a bit wrinkled and crumpled. The roughness of the surface was likely attributed to the stacking of ultrathin graphene sheets in the dry state and the coating layer consisting of PtNi particles (Figure 3A). On the other hand, the presence of PANI led to a significantly rougher surface formed by the stacked PANI nano-spheres (an average diameter of 70 nm) on the rGO surface in both rGO/PANI@PtNi and rGO/PANI composites (Figure 3B and Figure S7).

**Figure 4:**
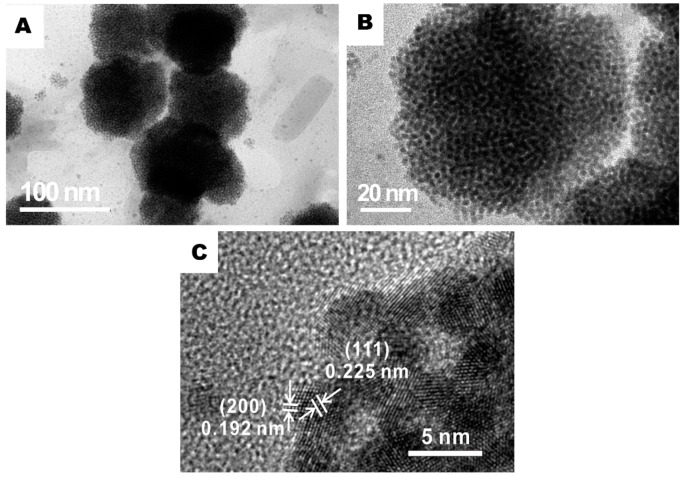
Representative high revolution transmission electron microscope (HRTEM) images of the rGO/PANI@PtNi composite at various magnifications: (**a**) ×75,000; (**b**) ×195,000; (**c**) ×1,000,000

The distribution of PtNi particles in rGO/PANI@PtNi were examined by high revolution transmission electron microscope (HRTEM). Figure 4 indicates that the vast majority of the PtNi particles thoroughly wrapped the PANI spheres monodispersely. This selective location of the metallic particles could be attributed to the stronger attraction from amine groups to the metallic ions than the one from hydroxyl and carboxyl groups on the rGO surface [50]. The d values for the metal crystal plane (111) were estimated with a value of 0.225 nm, which was slightly smaller than the one of pristine Pt (111) crystal planes (0.227 nm) [51,52]. On the other hand, the d spacing for (200) was 0.192 nm, which could be assigned to the (200) plane of Pt3Ni alloy (0.191 nm) [53]. Considering this crystallographic plane, it might conclude an alloy formation between Pt and Ni on the PANI surface after microwave reduction. Additionally, the presence of PANI was also found to be beneficial to control the particle size. The average diameter of PtNi was sharply reduced from 8.1 nm for rGO/PtNi to 1.7 nm for rGO/PANI@PtNi with an apparently narrower distribution (Figure S8). This superior dispersibility on PANI and well-controlled particle size of PtNi particles could have arisen from the tremendously large active area for anchoring the metallic precursors, which was provided by the well-stacked PANI-nanoparticles with a small average diameter of only 70 nm. 

**Figure 5:**
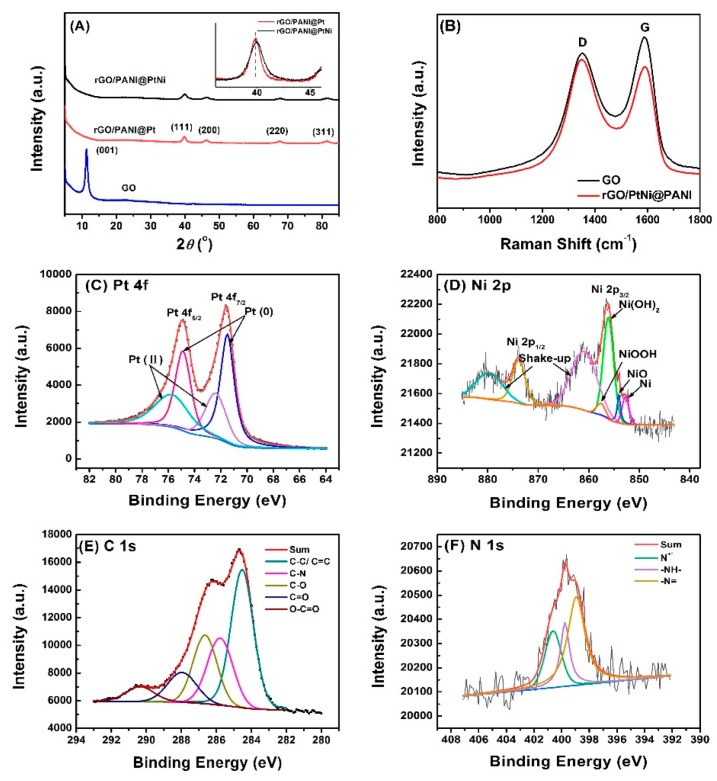
XRD patterns of the as-synthesized GO, rGO/PANI@PtNi, and rGO/PANI@Pt. Inset: the amplified plots of (111) peaks for rGO/PANI@PtNi and rGO/PANI@Pt (**A**); Raman spectroscopy of GO and rGO/PtNi@PANI nanocomposite (**B**); X-ray photoelectron spectroscopy (XPS) of Pt 4f (**C**), Ni 2p (**D**), C 1s (**E**), and N 1s (**F**) for the rGO/PANI@PtNi composite.

To further understand the structure of the composites, rGO/PANI@PtNi was further characterized by XRD (Figure 5A), Raman spectroscopy (Figure 5B), as well as XPS (Figure 5C–F). The XRD pattern of GO exhibited the strong (001) peak at 11.3°, while it vanished in the spectrum of the rGO/PANI@PtNi sample, confirming successful reduction of GO after the microwave treatment [5,54]. Moreover, the presence of PANI@PtNi nano-spheres was found to serve as spacers to prevent the graphene sheets from restacking due to van der Waals interactions. Therefore, the increased interlayer between the rGO sheets was another factor leading to the disappearance of the (001) rGO diffraction peak [5,55]. The peaks at 39.9°, 46.3°, 67.8°, and 81.5° of the rGO/PANI@PtNi composite could be assigned to (111), (200), (220), and (311) of fcc structured Pt nanocrystals, respectively [56]. However, there were no characteristic peaks of Ni or its oxides/hydroxides detected in the composites. This could be explained by the formation of alloy between Pt and Ni, since Pt is known to be alloyed well with Ni [57]. Furthermore, the 2θ value of the (111) peak in the case of rGO/PANI@PtNi (39.9°) exhibited a higher angle shift than that of the rGO/PANI@Pt without Ni (39.7°), which also accounted for the alloy of Pt and Ni [51,58,59,60]. Thus, XRD results were in good accordance with the crystallographic plane presented in HRTEM results and verified the successful alloy formation of PtNi in the composite. 

The reduction of GO into rGO through microwave treatment was further identified by comparing Raman spectrum of GO and rGO/PANI@PtNi (Figure 5B). Both of the curves showed obvious peaks at ~1350 cm^−1^ and 1589 cm^−1^, corresponding to the D band and the G band, respectively. The D band was attributed to the disorder and the imperfection of the carbon crystallites, while the G band originated from the in-plane bond-stretching vibration of sp2 bonded. Thus, the intensity ratio of the D/G bands (I_D_/I_G_) reflected the extent of sp2 domains and structural disorder of graphene sheets. In this work, although the rGO/PANI@PtNi composite was prepared via a reduction processing, its I_D_/I_G_ (0.71) was almost two times that of the ratio of GO (1.44), illustrating the decreased in-plane sp^2^ domains and a partially ordered crystal structure of the reduced sheets. This relatively high I_D_/I_G_ in rGO/PANI@PtNi was in good accordance with the results reported in literature about rGO composites and could be explained by the limited size of the reestablished rGO network (sp^2^ carbon) relative to the original ones, as well as the interactions among the graphene, the PANI, and the PtNi [22,25,28].

The chemical states of rGO/PANI@PtNi composites were detected by XPS that clearly demonstrated the synergic combination of all three components (Figure S9). The fitted spectra of Pt 4f, N 2p, C 1s, and N 1s are shown in Figure 5C–F, respectively. In the spectrum of Pt 4f, the peaks with binding energies at 71.49 eV and 74.87 eV for Pt (0) (Pt 4f_7/2_ and Pt 4f_5/2_) and 72.38 eV and 75.8 eV for Pt (II) (Pt 4f_7/2_ and Pt 4f_5/2_) were detected, respectively. The content of Pt (0) (60%) was higher than that of Pt (II) (40%). Moreover, with the reference to rGO/PANI@Pt, the Pt 4f_7/2_ and the Pt 4f_5/2_ peaks of rGO/PANI@PtNi displayed a very obvious shift from 72.73 eV and 75.97 eV for rGO/PANI@Pt to 72.43 and 75.72 eV for rGO/PANI@PtNi (Figure S10), which was ascribed to the electron donating from Ni sites occurring in the PtNi alloy [56,60]. On the other hand, the Ni 2p spectrum indicated the existence of metallic oxides and hydroxide rather than Ni (0) state, which was found to be universal in the case of PtNi alloys [56,61,62]. In detail, the Ni 2p_3/2_ XPS at the binding energies of 853.4 eV, 856.2 eV, and 857.3 eV were supposed to be assigned to NiO, Ni(OH)_2_, and NiOOH, respectively [60]. These XPS results for the amorphous Ni could well explain that peaks of Ni were not detected in XRD spectrum of rGO/PANI@PtNi (Figure 5A). Besides, the maintenance of the doped state for PANI spheres was also detected by the peak at the binding energy higher than 400 eV (400.6 eV) in the curve-fitted N 1s spectrum (Figure 5F), which was very essential for the composite to acquire good conductivity [63]. From the above results of XRD, XPS, and Raman, it could be concluded that the reduction of GO and the formation of PtNi alloy with hydrogen groups successfully occurred via the microwave fabrication in the case of rGO/PANI@PtNi, which were expected to facilitate the electrochemical performance of the rGO/PANI@PtNi composite in view of its potential superiority in electrical conductivity and catalysis.

### 3.3. Electrochemical Properties of the Modified GCE

**Figure 6:**
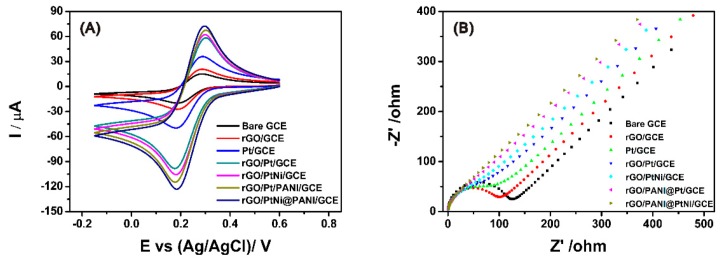
Cyclic voltammetry (CV) of bare GCE, rGO/GCE, Pt/GCE, rGO/Pt/GCE, rGO/PtNi/GCE, rGO/PANI@Pt/GCE and rGO/PANI@PtNi/GCE rGO/PtNi/GCE, rGO/PANI@Pt/GCE and rGO/PANI@PtNi/GCE in 0.1 M KCl containing 5.0 mM Fe(CN)_6_^3−^ at the scan rate of 50 mV/s (**A**); electronic impedance spectroscopy (EIS) spectra obtained at bare GCE, rGO/GCE, Pt/GCE, rGO/Pt/GCE, rGO/PtNi/GCE, rGO/PANI@Pt/GCE and rGO/PANI@PtNi/GCE in 0.1 M KCl containing 5.0 mM Fe(CN)_6_^3−/4−^ (**B**).

The conductivities of the different electrodes were characterized by carrying out the cyclic voltammetry (CV) on the different electrodes in 0.1 M KCl solution containing 5.0 mM K_3_Fe(CN)_6_^3−^. As shown in Figure 6A, a typical quasi-reversible one-electron redox behavior of ferricyanide ions was observed in the curves of all the electrodes. The influence of each component on the electro-conductivity of the composites was investigated through comparing the peak current and the average electroactive surface area of the different electrodes (Table 1). The average electroactive surface area (*A)* was estimated based on the Randles–Sevcik equation [64]:

(1) Ip=2.69×105×A×D12×n23×γ12×C

where *Ip* is the redox peak current, *A* relates to the area of the electroactive surface area (cm^2^), the diffusion coefficient of the molecule in solution (*D*) is 6.70 ± 0.02 × 10^−6^ cm^2^ s^−1^, *n* is the number of electrons participating in the redox reaction (*n* = 1 in case of one-electrode here), *γ* is the scan rate of the potential perturbation (0.05 V s^−1^), and *C* corresponds to the bulk concentration of the redox probe (5 mM). With reference to the bare GCE, the modification of GCE with rGO exhibited a very limited improvement on the conductivity only with an increase of 5.7 µA. After Pt particles were loaded on the rGO surface, the peak current of the rGO/Pt significantly increased; meanwhile, ∆*E_p_* became 23 mV smaller than the one of rGO/GCE, indicating the importance of Pt to improve the conductivity of the graphene materials. Moreover, PANI was also found to further facilitate the improvement of the conductivity with the increase of anode current both from 58.3 µA for rGO/Pt to 67.4 µA for rGO/PANI@Pt and from 62.0 µA for rGO/PtNi to 72.4 µA for rGO/PANI@PtNi. With PANI spheres, the electroactive surface area could be increased for more than 0.012 cm^2^. Importantly, the electroactive surface area of rGO/PANI@PtNi was 4.8 times that of bare GCE and 3.4 times that of pristine rGO. However, we also noticed that the enhancement resulting from the addition of Ni was very slight (less than 10 µA) as compared with the effects of Pt and rGO, which could be explained by the amorphous state of Ni in the PtNi alloy. Thus, we strongly suggest that Pt and PANI were supposed to display the predominant contribution to the promotion of the electrical conductivity for the rGO/PANI@PtNi composite rather than the Ni. The results from EIS (Figure 6B) were in good accordance with this conclusion with an electron transfer resistance (*R_ct_*) in sequence of: *R_ct-bare_* > *R_ct-rGO_* > *R_ct-Pt_* > *R_ct-rGO/Pt_*> *R_ct-rGO/PtNi_* > *R_ct- rGO/PANI@Pt_* > *R_ct-rGO/PANI@PtNi_*.

### 3.4. Sensing Performance of the Modified Electrodes for H_2_O_2_ Detection

The amperometric response of the modified GCE for H_2_O_2_ was determined in 0.2 M N_2_-saturated PBS solution containing 0.1 mM H_2_O_2_ at a scan rate of 50 mV/s with a successive addition of H_2_O_2_. In order to acquire efficient sensitivity and quantitative electrochemical detection, the pH value for testing was optimized by characterizing the sensing performance of the rGO/PANI@PtNi/GEC in the test solution with various pH values (Figure S11). Based on the resultant curves, an optimal solution pH of 6.5 was detected in view of its highest peak current, which was applied in the following electrochemical characterizations. 

**Figure 7:**
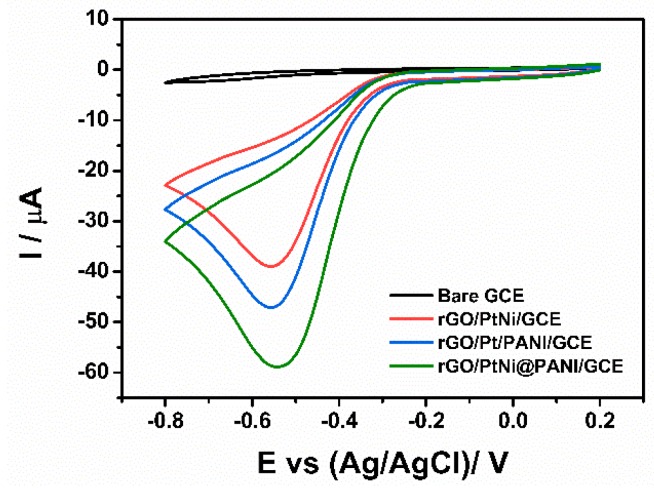
CVs of bare GCE, rGO/PtNi/GCE, rGO/Pt/PANI/GCE, and rGO/PANI@PtNi/GCE in N_2_-saturated 0.2 M phosphate buffered saline (PBS) at pH: 6.5 in the presence of 0.1 mM H_2_O_2_ (scan rate: 50 mV/s).

Figure 7 gives the cyclic voltammograms of bare GCE, rGO/PtNi/GCE, rGO/Pt/PANI/GCE, and rGO/PANI@PtNi/GCE being detected at pH = 6.5. In the case of the bare GCE, both the oxidation and the reduction peaks were barely visible, which could be explained by the extremely slow reaction process occurring on the electrode without any modification. Similar to the previous study of the composite sensor based on rGO and Pt particles [28,65], a reduction peak could be distinctly observed in the potential window (−0.8 V to 2.0 V) for all the electrodes containing Pt sites, while no oxidation peak appeared in the reverse potential scan. Thus, it could be concluded that the H_2_O_2_ detection on the modified electrodes underwent an irreversible reduction process. Among the modified electrodes, the rGO/PANI@PtNi/GCE displayed the highest current response and also the most positive reduction potential, illustrating its outstanding electronic transfer for the H_2_O_2_ testing. Comparing the response of rGO/PANI@PtNi/GCE with the one of rGO/PtNi/GCE, PANI nano-spheres showed noteworthy effects in increasing the cathodic peak current for the H_2_O_2_ testing system from 39.2 µA for rGO/PtNi/GCE to 59.0 µA for rGO/PANI@PtNi/GCE, which was in accordance with the conductivity tested in the [Fe(CN)_6_^3−^] solution discussed before. Furthermore, it was also interesting to note that the addition of Ni led to a relatively higher amplification of current response of 11.8 µA from GO/PANI@Pt/GCE to rGO/PANI@PtNi/GCE.

These results could be explained by the facilitation from Ni hydroxides for the electrocatalytic property of Pt. Based on the previous systematical studies about the mechanism of H_2_O_2_ electrochemistry on Pt particles [66,67], the electrochemical decomposition of H_2_O_2_ on Pt firstly started with the dissociation of H_2_O_2_ into OH groups followed by absorbing on at least two Pt sites’ surfaces:

(2) $$\mathrm{Pt}+H_{2}O_{2}\to 2\mathrm{Pt}\left(\mathrm{OH}\right)$$

Then, an electrochemical reduction of the resultant Pt(OH) subsequently occurred, being initiated by the instable absorbed OH on the Pt sites:

(3) $$4\mathrm{Pt}\left(\mathrm{OH}\right)\overset{e^{-}}{\to }4\mathrm{Pt}+2H_{2}O+O_{2}$$

Owing to this regeneration, the free Pt sites were formed again and available for the next dissociation of other H_2_O_2_. From XPS spectrum of Ni 2p for rGO/PANI@PtNi (Figure 5D), it was suggested that the surface layer of PtNi alloy contained both Ni(OH) and NiOOH. In several former works [60,61,68,69], such mixed-valence Ni^2+^/Ni^3+^ has been demonstrated to be advantageous to improve the conductivity and the catalytic activity through the following electrochemical reversible reaction:

(4) $$\mathrm{Ni}{\left(\mathrm{OH}\right)}_{2}↔\mathrm{NiOOH}+H^{+}+e^{-}$$

**Figure 8:**
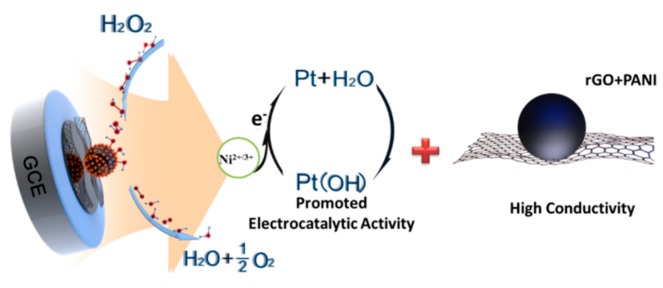
Scheme of the mechanism of H_2_O_2_ decomposition on rGO/PANI@PtNi/GCE.

Thus, forming alloy with Ni could effectively promote the catalytical efficiency of Pt sites with high current response for H_2_O_2_. Considering the above electrochemical characterizations, we deemed that the facilitated electrochemical sensitivity of rGO/PANI@PtNi for H_2_O_2_ was attributed to two synergistic factors (Figure 8). First, the good stacking of the doped-PANI nano-spheres on the rGO surface could be beneficial to improving the electronic conductivity in the system, not only resulting from their own superior conductivity but also from the sophisticated structure they formed with a large active area for both electronic transfer and absorption of the catalyst. Second, the existence of Ni in the PtNi alloy efficiently facilitated the electrocatalytic performance of the composite by promoting the electrochemical reduction of the regeneration of Pt sites during the H_2_O_2_ decomposition. 

**Figure 9:**
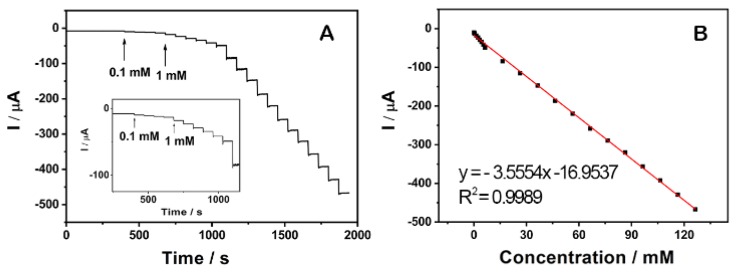
Typical steady-state response of the rGO/PANI@PtNi GCE to successive injection of H_2_O_2_ into the N_2_-saturated 0.2 M PBS with pH = 6.5 at 0 V. Inset: the amplified response curve at low concentrations (**A**); the corresponding calibration curve at 0 V (**B**).

Figure 9A shows a typical current-time plot of the rGO/PANI@PtNi modified GCE for H_2_O_2_ detection by successively adding H_2_O_2_ into the stirring PBS (pH = 6.5) under an applied voltage of 0 V. It clearly reveals that the current response increased with the concentration of H_2_O_2_, and the sensor rapidly achieved 96% of the steady state current in 2 s. The sensitivity of rGO/PANI@PtNi was further characterized by studying the calibration plot of the sensor, as shown in Figure 9B. The linear detection range was estimated to be from 0.1 mM to 126 mM (R^2^ = 0.9989), and the detection limit was estimated to be 0.5 µM on the basis of S/N = 3 [22,70]. This detection limitation was much lower than that of the rGO/PANI@Pt/GCE (1.1 µM) without the addition of Ni (Figure S12), which further confirmed the positive effect of Ni sites on facilitating the electrocatalytic performance. The sensitivity and the detection limit of rGO/PANI@PtNi/GCE showed favorable and competitive electrocatalytic performance compared to that of some previously reported sensors based on graphene sheets (Table 2). It could therefore be suggested that rGO/PANI@PtNi/GCE possessed a superior sensitivity for H_2_O_2_ detection, which could be attributed to a synergistic effect combining the improved electron transfer and the promoted electrocatalytic property as well as the large active area originating from the good assembly of PANI nano-spheres on rGO sheets.

Furthermore, the long-term stability of the rGO/PANI@PtNi modified electrode was also evaluated by carrying out the cyclic voltammogram under the same conditions after seven days (Figure S13). During the experiment, the used rGO/PANI@PtNi/GCE was preserved in air at room temperature. The cathodic peak current of this stored sample for seven days was found to have good stability only with a slight current decrease of 0.51%, demonstrating the composite possessed not only a good electrochemical behavior for H_2_O_2_-detection but also sufficient environmental stability for long-term use. 

## 4. Conclusions

In this paper, we fabricated a novel hierarchical rGO/PANI@PtNi nanocomposite with PtNi decorated PANI nano-spheres well stacked on the rGO sheet. Using water-stable PANI nano-sphere suspensions effectively inhibited the stacking of graphene sheets and the aggregation of nano-catalyst, leading to well size-controlled PtNi particles with quite a small diameter (1.7 ± 0.1 nm). Moreover, the PANI nano-spheres on the rGO sheet generated a remarkably rougher surface compared to the composites without PANI, providing a large active surface area for both the electronic transfer and the anchoring of the precursor for the electrocatalysts. Consequently, rGO/PANI@PtNi/GCE exhibited conspicuous enhancement on the electronic conductivity by adding PANI. Importantly, rGO/PANI@PtNi/GCE also displayed high conductive and electrocatalytic properties for H_2_O_2_ detection. A linear dependence of the biosensor signals was detected from concentrations between 0.1 mM and 126 mM, and the detection limit of the sensor was 0.5 µM (S/N = 3). In addition to the increased electro-conductivity, the promoted electrocatalytic property of Pt sites by the multi-valance Ni^2+^/^3+^ in PtNi alloy was considered to be the other significant factor to upgrade the sensitivity for H_2_O_2_ decomposition. The synergic effect of the improved conductivity from PANI and rGO with a sophisticated structure and the promoted electrocatalytic property of PtNi alloy impart a high electrochemical sensitivity to rGO/PANI@PtNi/GCE and offer a promising approach for the development of the nonenzymatic sensors towards H_2_O_2_ detection.

## Figures and Tables

**Table 1:** The anodic peak current and the average electroactive surface area of PtNi on different electrodes.

Electrode	Anodic Peak Current (µA)	Average Electroactive Surface Area * (A) (cm^2^)
Bare GCE	15.0	0.019
rGO/GCE	20.7	0.027
Pt/GCE	35.8	0.046
rGO/Pt/GCE	58.3	0.075
rGO/PtNi/GCE	62.0	0.079
rGO/PANI@Pt/GCE	67.4	0.087
rGO/PANI@PtNi/GCE	72.4	0.093

* Average electroactive surface area was calculated based on the Equation (1), as discussed above. The geometry of the bare electrode was a disk with a diameter of 3 mm (the geometric area was 0.071 cm^−2^).

**Table 2:** Comparison of the electrochemical performance of H_2_O_2_ sensors based on the graphene composites.

Material	Applied Potential (V)	Linear Range (μM)	LOD (μM)	Ref
Graphene-Pt	0	2–710	0.5	[71]
Catalase/Porous Graphene	−0.35	0.1–7.7	0.083	[72]
Graphene-CNT-Pt	−0.005	0.1–25	0.01	[73]
PtNPs/Graphene paper	−0.25	0.2–2000	0.1	[74]
Fe_3_O_4_@PANI/rGO	−0.9	100–1500	4.45	[75]
AgNPs/N-doped graphene	−0.3	100–126,400	1200	[5]
RGO/nAPAMSs (AuPt alloy)	−0.5	5–4000	0.008	[76]
Nf/Pd@Ag/rGO-NH_2_/GCE	−0.45	2–19,500	0.7	[77]
Ag/F-SiO_2_/GO	−0.3	100–260,000	4	[78]
Cu_2_O/PANI/rGO	−0.1	0.8–12,780	0.5	[79]
Au-graphene-HRP-chitosan	−0.25	20–8000	1.7	[80]
Au@PB NP graphene paper	−0.1	1–30	0.1	[81]
rGO/PANI@Pt	0	100–126,400	1.1	This work
rGO/PANI@PtNi	0	100–126,400	0.5	This work

## Supplementary Materials

The following are available online at https://www.mdpi.com/2079-4991/9/8/1109/s1. Figure S1: TEM image of rGO, Figure S2: TEM images of platinum nanoparticles, Figure S3: TEM image of rGO/Pt nanocomposite, Figure S4: TEM image of rGO/PtNi nanocomposite, Figure S5: SEM of rGO/PANI@Pt nanocomposite, Figure S6: FT-IR spectrum of PANI, Figure S7: TEM and SEM images of rGO/PANI, Figure S8: Representative TEM images and the corresponding histograms of the particle size distribution of the PtNi metallic nanoparticles distributed on the surface of rGO/PtNi and rGO/PANI@PtNi, Figure S9: XPS Spectrum: wide survey spectra of the rGO/PANI@PtNi nanocomposite, Figure S10: XPS spectrum of Pt 4f from the rGO/PANI@Pt and rGO/PANI@PtNi composite, Figure S11: Cyclic voltammograms of rGO/PANI@PtNi/GCE in N_2_-saturated 0.2 M PBS at different pH value in the presence of 0.1 mM H_2_O_2_, Figure S12: Typical steady-state response of the rGO/PANI@Pt/GCE to successive injection of H_2_O_2_ and the corresponding calibration curve, Figure S13: CVs of pristine rGO/PANI@PtNi/GCE and the rGO/PANI@PtNi/GCE stored for 7 days.
