# Supplementary material for: Facile Fabrication of Hierarchical rGO/PANI@PtNi Nanocomposite via Microwave-Assisted Treatment for Non-Enzymatic Detection of Hydrogen Peroxide
Source: Nanomaterials (Basel). 2019 Aug 2;9(8):1109. doi: 10.3390/nano9081109 (PMC6722818; doi:10.3390/nano9081109)
Supplement: Supplementary file 1 [file nanomaterials-09-01109-s001.pdf]

Electronic Supporting Information (ESI)

Facile fabrication of hierarchical rGO/PANI@PtNi nanocomposite via microwave-assisted treatment for non-enzymatic detection of hydrogen peroxide

*Fa-Gui He [1,2], Jia-Yi Yin [1], Gaurav Sharm [1,2], Amit Kumar [1,2], Florian J. Stadler [1] and Bing Du [1,] **

[1] College of Materials Science and Engineering, Shenzhen Key Laboratory of Polymer Science and Technology, Guangdong Research Center for Interfacial Engineering of Functional Materials, Nanshan District Key Lab for Biopolymers and Safety Evaluation, Shenzhen University, Shenzhen 518055, PR China; hepengzhuo@foxmail.com (F.-G.H.); Amy_YinJY@163.com (J.-Y.Y.); gaurav8777@gmail.com (G.S.); mittuchem83@gmail.com (A.K.); fjstadler@szu.edu.cn (F.J.S.);

[2] Department of Optoelectronic Engineering, Shenzhen University, Shenzhen 518060, Guangdong, China.

* E-mail: dubing@szu.edu.cn

Table of Contents

Figure S1. TEM image of rGO.	S3
Figure S2. TEM images of platinum nanoparticles.	S4
Figure S3. TEM image of rGO/Pt nanocomposite.	S5
Figure S4. TEM image of rGO/PtNi nanocomposite.	S6
Figure S5 SEM of rGO/PANI@Pt nanocomposite.	S7
Figure S6. FT-IR spectrum of PANI.	S8

Figure S7. TEM and SEM images of rGO/PANI.	S9
Figure S8. Representative TEM images and the corresponding histograms of the particle size distribution of the PtNi metallic nanoparticles distributed on the surface of rGO/PtNi and rGO/PANI@PtNi.	S10
Figure S9. XPS Spectrum: wide survey spectra of the rGO/PANI@PtNi nanocomposite.	S11
Figure S10. XPS spectrum of Pt 4f from the rGO/PANI@Pt and rGO/PANI@PtNi composite.	S12
Figure S11. Cyclic voltammograms of rGO/PANI@PtNi/GCE in N_2-saturated 0.2 M PBS at different pH value in the presence of 0.1 mM H_2O_2.	S13
Figure S12. Typical steady-state response of the rGO/PANI@Pt/GCE to successive injection of H_2O_2 and the corresponding calibration curve.	S14
Figure S13. CVs of pristine rGO/PANI@PtNi/GCE and the rGO/PANI@PtNi/GCE stored for 7 days.	S15

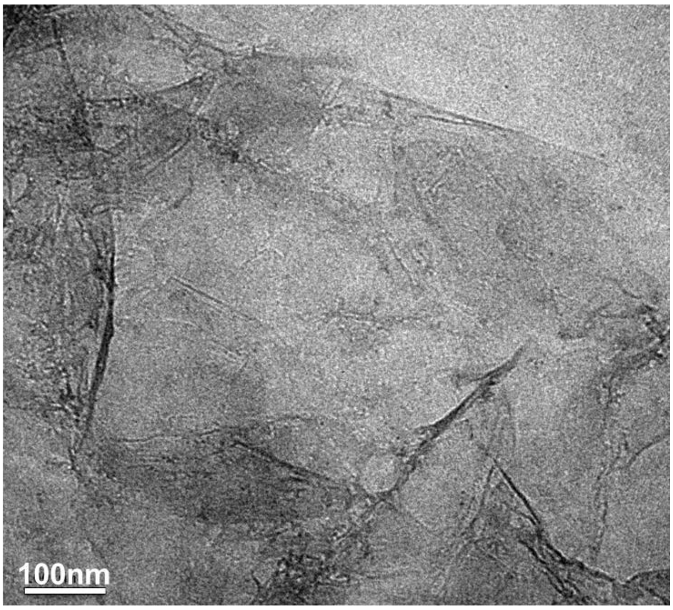

Figure S1. TEM image of rGO.

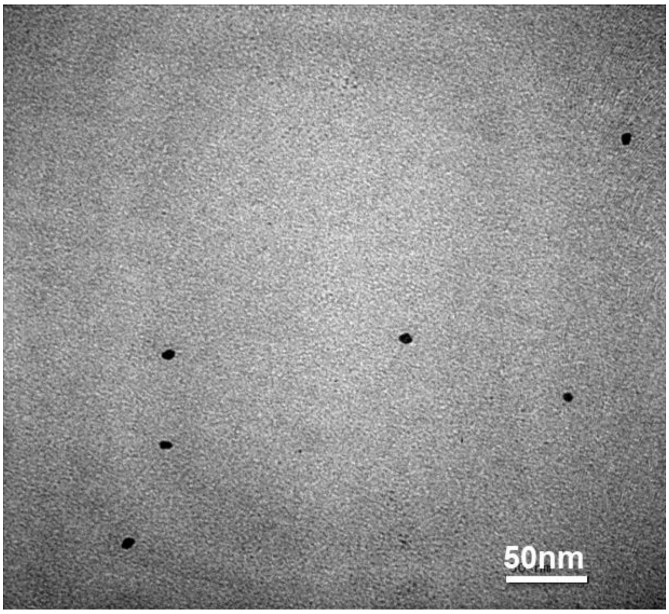

Figure S2. TEM images of platinum nanoparticles.

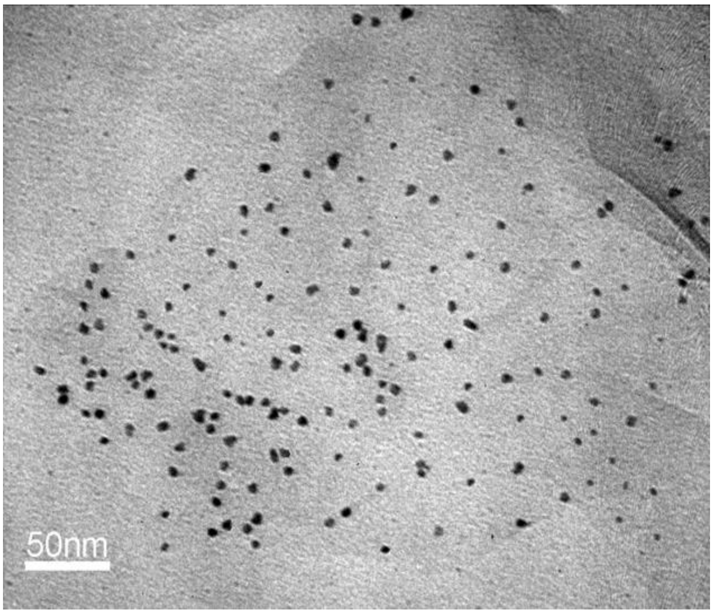

Figure S3. TEM image of rGO/Pt nanocomposite.

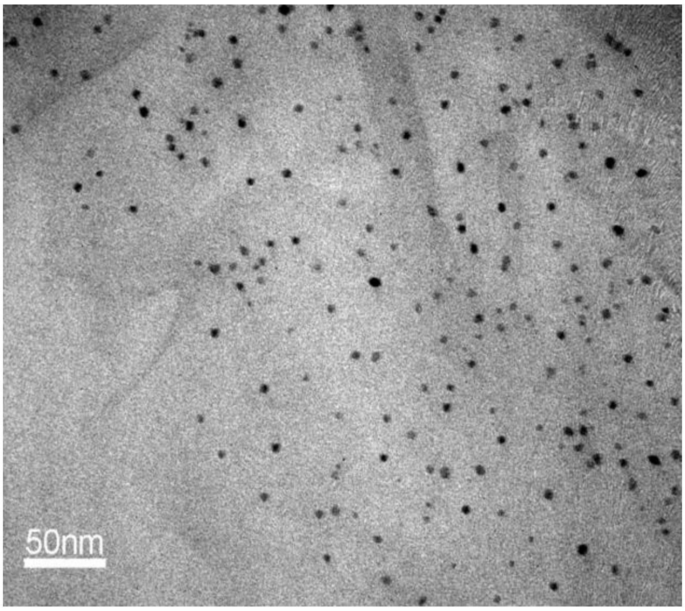

Figure S4. TEM image of rGO/PtNi nanocomposite.

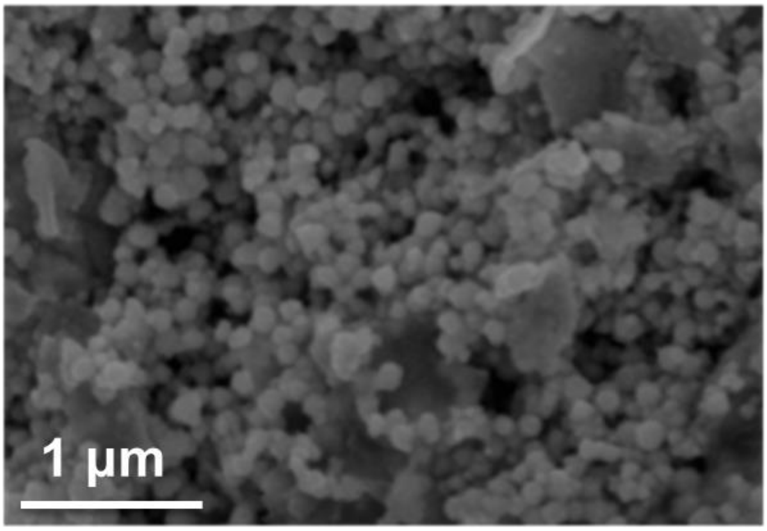

Figure S5. SEM of rGO/PANI@Pt nanocomposite.

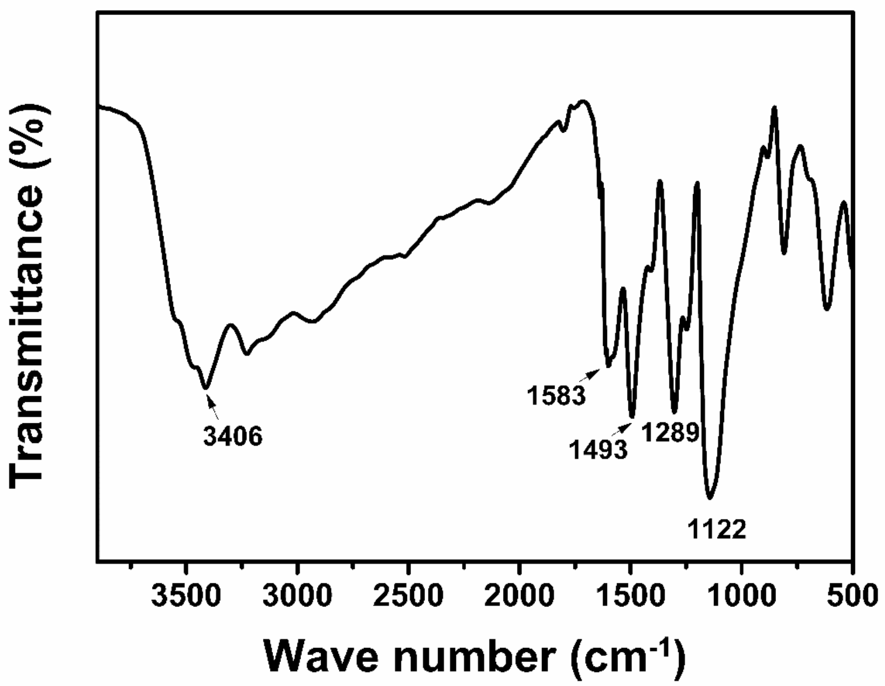

Figure S6. FT-IR spectrum of PANI.

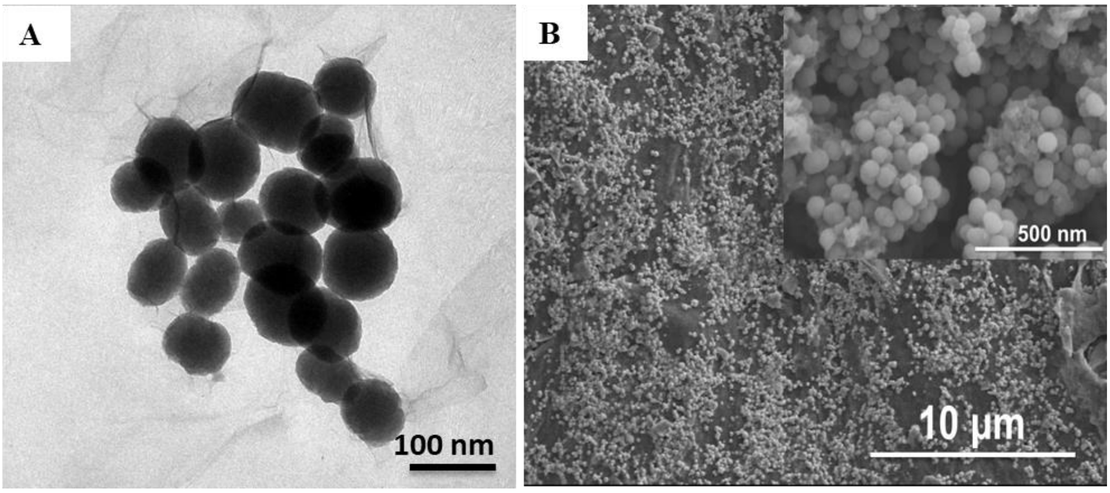

Figure S7. (A) TEM and (B) SEM images of rGO/PANI.

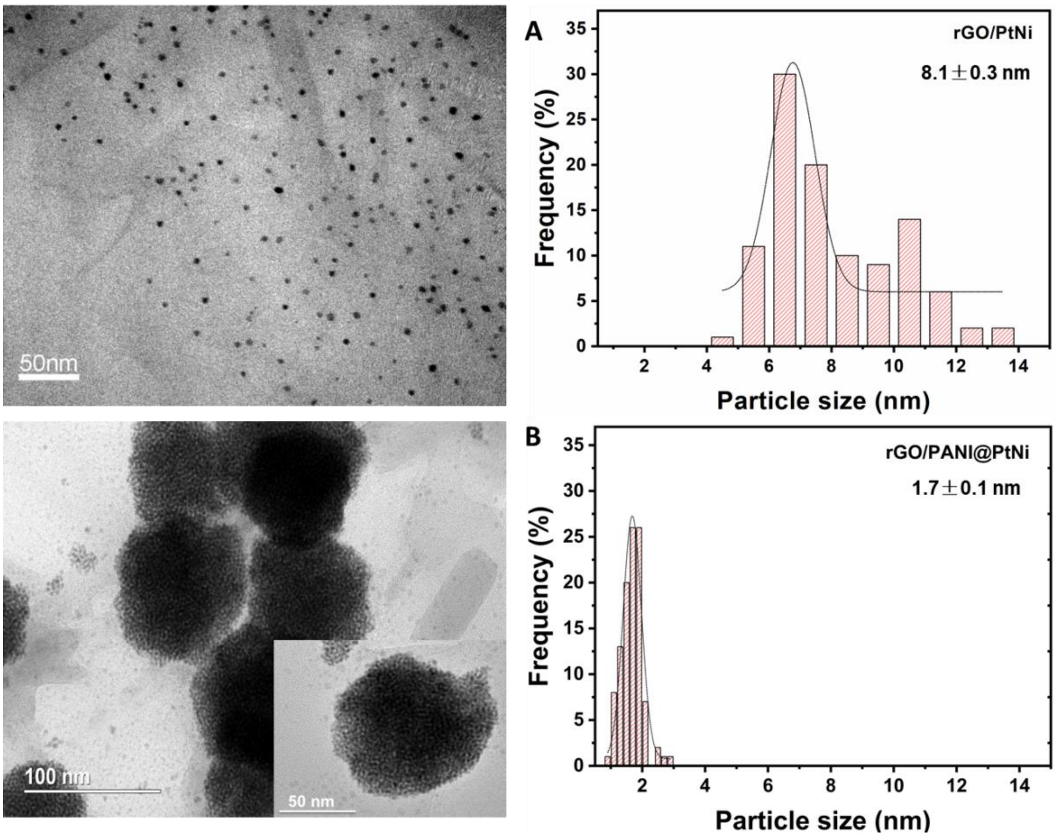

Figure S8. Representative TEM images (left) and the corresponding histograms of the particle size distribution (right) of the PtNi metallic nanoparticles distributed on the surface of A: rGO/PtNi; and B: rGO/ /PANI@PtNi.

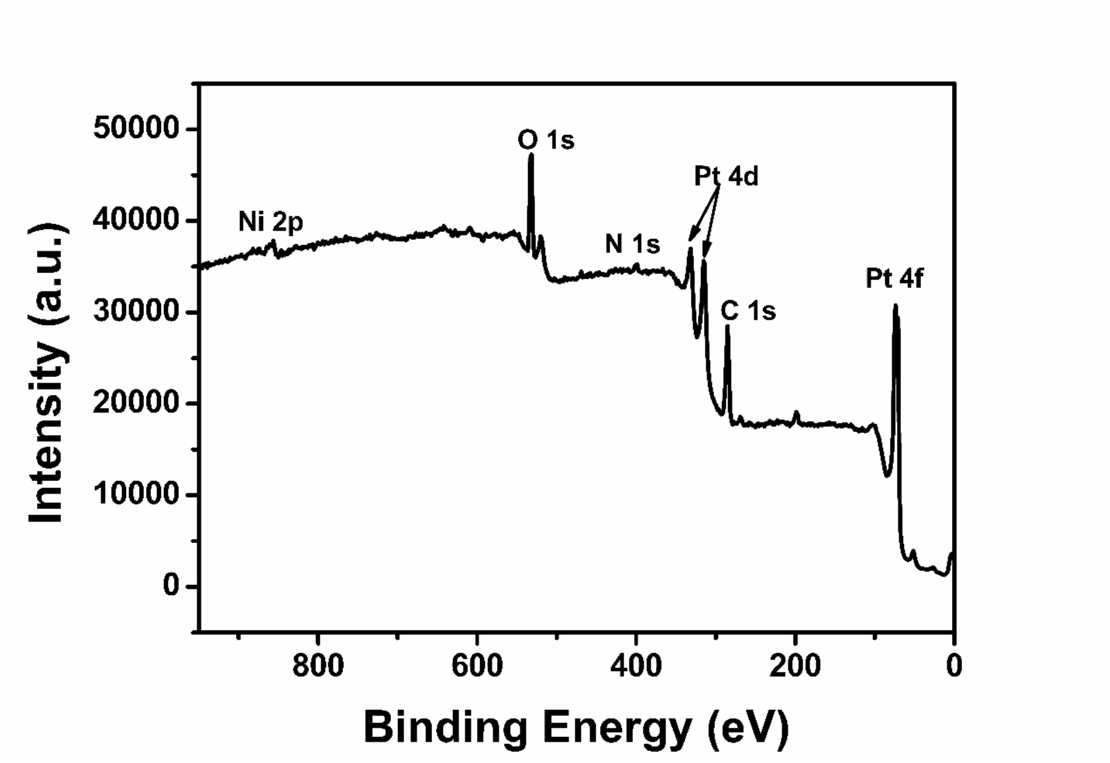

Figure S9. XPS Spectrum: wide survey spectra of the rGO/PANI@PtNi nanocomposite.

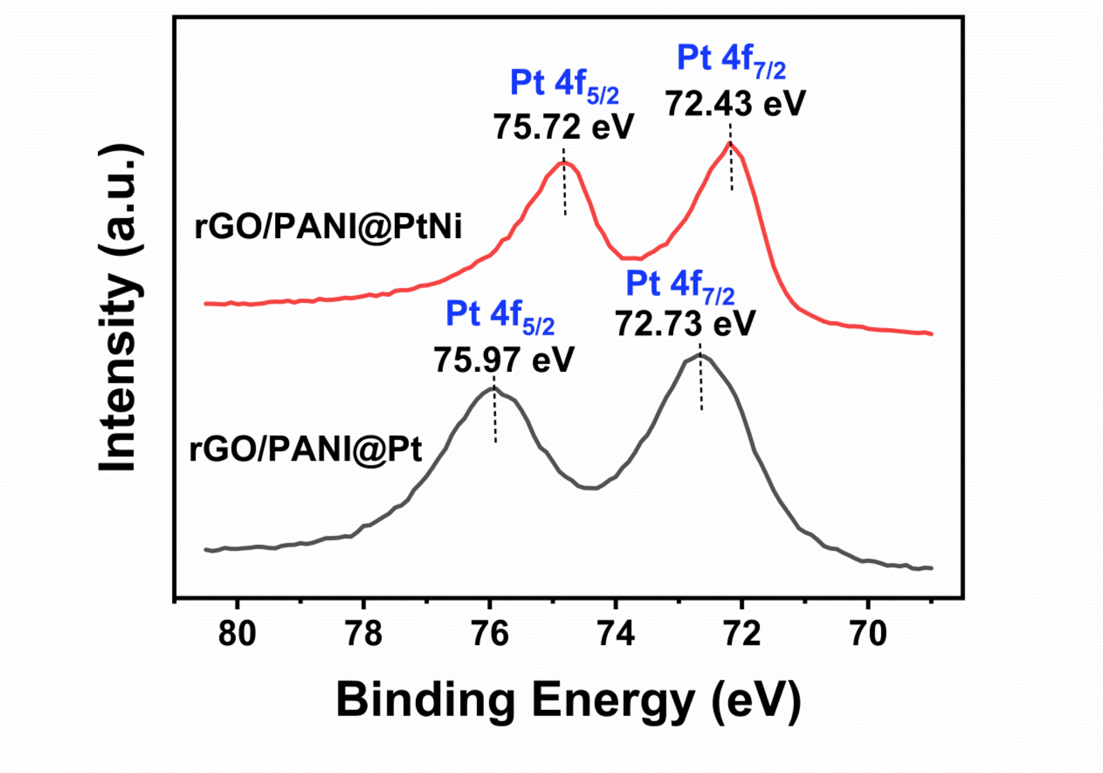

Figure S10. XPS spectrum of Pt 4f from the rGO/PANI@Pt and rGO/PANI@PtNi composite.

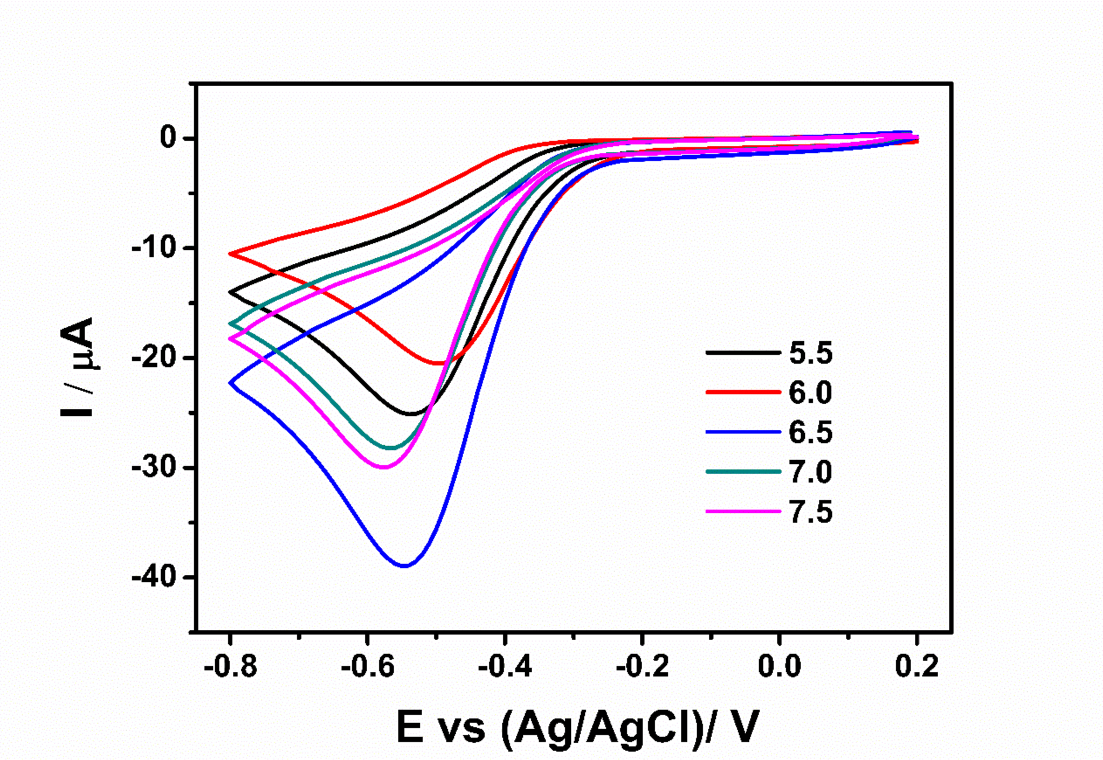

Figure S11. Cyclic voltammograms of rGO/PANI@PtNi/GCE in N_2-saturated 0.2 M PBS at different pH value in the presence of 0.1 mM H_2O_2 (scan rate: 50 mV/s).

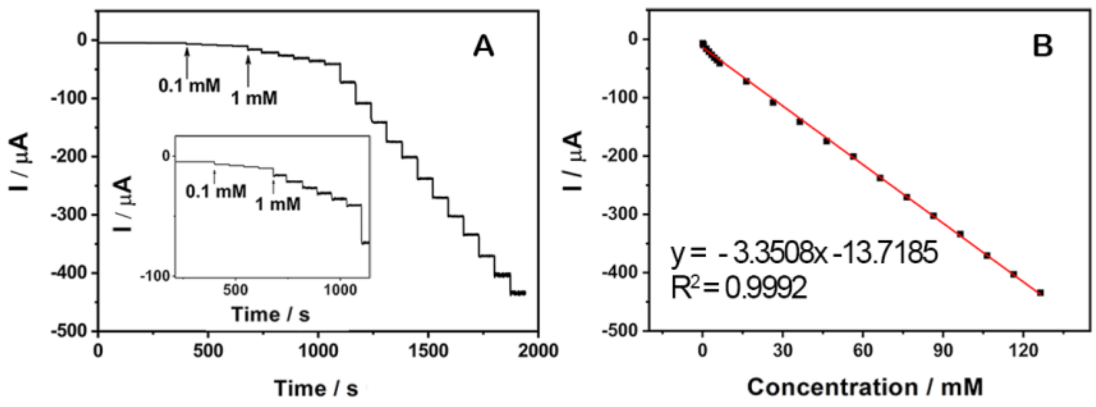

Figure S12. (A): Typical steady-state response of the rGO/PANI@Pt/GCE to successive injection of H_2O_2 into the N_2-saturated 0.2 M PBS at pH: 6.5. Inset: amplified response curve at low concentrations and (applied potential: 0V); (B): The corresponding calibration curve (applied potential: 0 V).

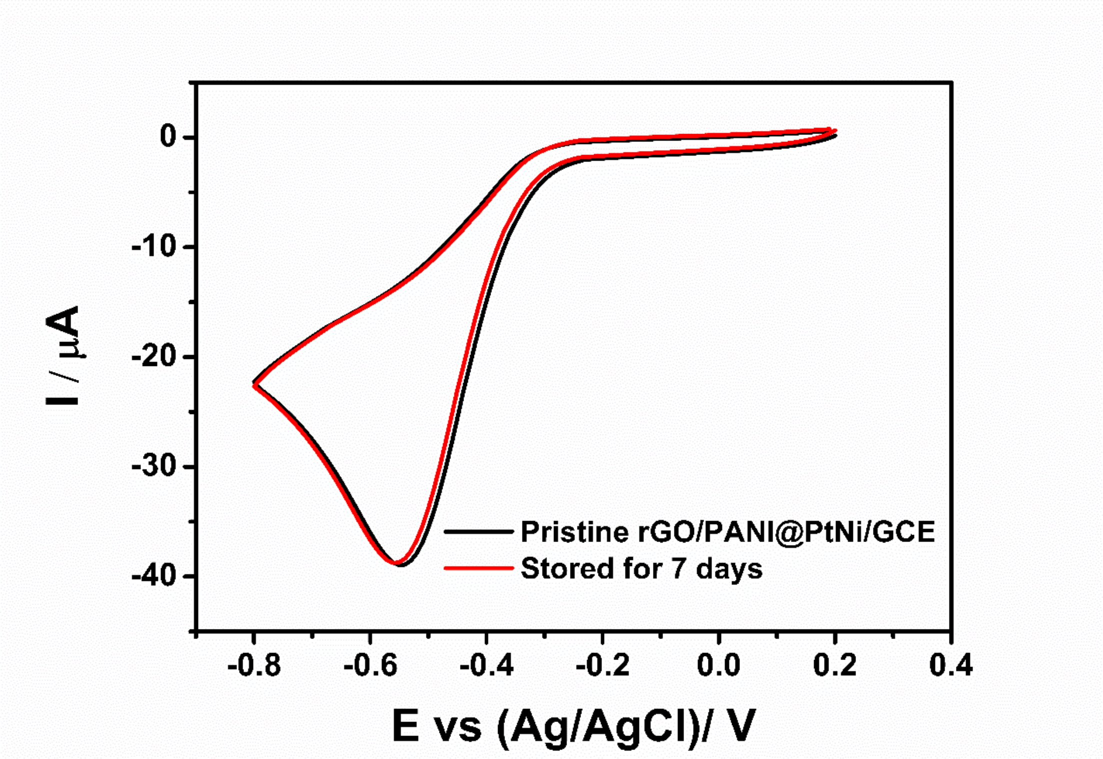

Figure S13. CVs of pristine rGO/PANI@PtNi/GCE and the rGO/PANI@PtNi/GCE stored for 7 days (N_2-saturated 0.2 M PBS, pH=6.5; 0.1 mM H_2O_2; scan rate: 50 mV/s).
